# Organisational Actions for Improving Recognition, Integration and Acceptance of Peer Support as Identified by a Current Peer Workforce

**DOI:** 10.1007/s10597-023-01179-x

**Published:** 2023-08-18

**Authors:** Verity Reeves, Mark Loughhead, Matthew Anthony Halpin, Nicholas Procter

**Affiliations:** The University of South Australia (Clinical Health Sciences), GPO Box 2471, Adelaide, 5001 Australia

**Keywords:** Peer support, Mental health recovery, Organisational integration, Lived experience, Organisational change management

## Abstract

Recovery-orientated practice is crucial to mental health care services—consistently identified in policy, service delivery guidelines and national mental health action plans. An essential component to systems reform and the adoption of recovery-orientated practice is the inclusion of peer support workers as practice leaders to support shifting culture in mental health service delivery. Designated peer support roles operate as healthcare professionals who utilise their lived and living experience of mental health difficulty to support those on their recovery journey through mutual understanding of shared experience. This research sought to explore the experiences of peer support workers integrating into mental health teams and identify organisational actions to facilitate successful recognition, integration and acceptance by colleagues; therefore, promoting sustainability of the peer support role. Qualitative interviews were undertaken with 18 peer support workers employed across four Australian states within 12 different government and non-government organisations. Study findings reveal three key areas for organisational change with seven main themes to assist organisations to better facilitate the successful integration of peer support workers into mental health service teams. These included robust induction, training for existing staff, clear referral pathways into the service, consistent supervision and debriefing, leadership support, professional development pathways and involving peer workers through change processes. These themes were grouped into three key areas for change including preparation, process and structural changes, and cultural change actions for sustainability. This article makes recommendations for organisations to consider when implementing peer support roles into mental health services.

## Introduction

The principles of recovery-orientated practice in mental health services revolve around the understanding that each individual is different and should be encouraged and assisted towards living a flourishing life, have hope for the future and supported to make informed decisions surrounding their healthcare journey to recovery (Davidson et al., 2016). This understanding and ethic underpins recommended best practice for present-day mental healthcare in many Western countries. Essential to meeting recovery-orientated principles within services is the integration of lived experience peer support into service delivery and practice (Cook et al., 2012; Pitt et al., 2013; Slade et al., 2014). Increasingly, peer support workforces are being incorporated within mental health policies, national action plans and utilised in service delivery globally (United Nations High Commission, 2017; World Health Organization, 2019). The role of peer support workers is to utilise their lived and living experience of mental health issues to support others on their recovery journey through mutual understanding of shared experience (Byrne et al., 2021b; Chinman et al., 2008a; Mancini, 2018). This approach to support embodies the core elements of recovery practice, focusing on strengths, activating hope and optimism and channelling this energy to empower the individual to take control and responsibility for their recovery journey (Repper & Carter, 2011; Zeng & McNamara, 2021).

The growth of the peer support workforce in Australia is demonstrated through the increasing presence of these roles into mental health service delivery, finding an annual growth rate of 15.9% between 2015 and 2020 (Australian Institute of Health and Welfare, 2023) and increased financial investment from a government committed to developing a growing mental health workforce (Department of Health and Aged Care 2023). Previous studies have identified and demonstrated some benefits of incorporating peer workers in an individual’s personal recovery journey. These include, increased hope, growth in confidence and empowerment for recovery, improved outcomes from help seeking behaviour, reduced crisis resulting in hospitalisation and greater autonomy in addition to developing better social connections (Bellamy et al., 2017; Davidson et al., 2012; King & Simmons, 2018; Lawn et al., 2008). These benefits can improve outcomes for both individual’s receiving supports and peer workers themselves.

Since the introduction of designated lived experience and peer support roles within mental health services, it has faced significant challenges. Inconsistent or poor understanding of the peer support role, its purpose, practices and benefits to service users are often flagged as known barriers to successfully embedding peer support or lived experience roles into mental health service teams (Byrne et al., 2021c; Ibrahim et al., 2021; Mutschler et al., 2022; Vandewalle et al., 2016). This is compounded by reduced support from the employing organisation and fewer training opportunities which can lead to poor valuing of lived experience expertise (attitudinally and financially), limit willingness for collaboration between disciplines and see prejudicial and stigmatizing attitudes persist (Byrne et al., 2021c). Poor working conditions and the presence and persistence of such challenges impact peer workers ability to have meaningful impact within the system (Ahmed et al., 2015; Byrne et al., 2016; Salzer et al., 2013).

Although some benefits have been established, it is worth noting a lack of heterogeneity in peer support research with inconsistent modality and typology across studies. Despite this, the barriers and challenges faced by peer workers need to be recognized and addressed. This gives foundation to organisational change, including improvement of organisational preparedness and understanding, commitment and inclusion of peer support expertise, appropriate recruitment and induction to the workplace, role clarity and ongoing comprehensive supervision (Byrne et al., 2021a; Ibrahim et al., 2021; Vandewalle et al., 2016; Zeng & McNamara, 2021). Particularly, poor role clarity or a lack of organisational preparedness and understanding can lead to misunderstandings of the role and ultimately a lack of acceptance by colleagues prompting instances of “othering”, exclusion and stigma (Byrne et al., 2018a, 2018b; Chinman et al., 2019). Such challenges have been found to impact the ability of the peer support worker to effectively perform their role and feel like a valued member of the team (Byrne et al., 2019b).

This research gathers the experiences of peer workers entering designated support roles and integrating within multidisciplinary mental healthcare teams. Peer workers are in a unique position to reflect on these experiences and provide insight into priorities and actions for the future. This study seeks to build on existing knowledge and address identified limitations of implementation of peer support (Chinman et al., 2017; Farkas & Boevink, 2018; Mutschler et al., 2022) by identifying and contextualising actions to compliment organisational change and adoption of recovery-orientated frameworks. For the purpose of this study, successful integration refers to creating a workspace where peer support is openly embraced and supported in workforce development, where organisations are able to provide clarity and distinguish boundaries for peer roles to meet service user and organisational needs, rather than inserting peer workforces into existing service contexts (Loughhead et al., 2021). This is about fundamental change in generating recovery-orientated workplace culture, compliment organisational change and development and support the sustainability of peer support roles.

## Method

This study aimed to investigate peer workers experiences being integrated into mental health organisations and their perspectives on how organisations can improve recognition, integration and acceptance of peer support. This applied a qualitative descriptive study design, utilizing semi-structured individual interviews with peer workers currently employed in publicly accessible mental health services.

### Recruitment of Participants

Snowball and purposive sampling techniques recruited a total of 18 peer support workers currently employed within mental health services across Australia. Initial recruitment materials were advertised and distributed electronically via email and online through webpage and social media platforms. Materials provided detailed information regarding the purpose, scope and qualifying inclusion criteria for the research. Potential participants meeting inclusion criteria as detailed in Table 1, were encouraged to contact the research team to discuss any further questions and arrange participation. Participants’ experience within the industry ranged from 3 months to 20 years, however, were asked to reflect on most recent on-boarding experiences which ranged from 3 months to 2 years. See Table 2 for participant demographics.

**Table 1:** Inclusion and exclusion criteria

Inclusion criteria	Exclusion criteria
Individuals identifying as having lived experience of mental distress and are currently or recently (within the past 12 months) employed in a paid peer support position within mental health services. Or individuals identifying as carer for an individual with lived experience and employed in carer peer support roles	Participation is restricted to those who have worked in a peer support role or alongside a designated lived experience role within mental health services. Any participant under the age of 18 was not eligible for this study. Any participant who does not sign written consent to participate or chooses to withdraw their consent was also excluded from this study

**Table 2:** Participant demographics

	Number (*N*)	Percentage (%)
Gender		
Female	12	66
Male	6	33
Gender diverse/chose not to disclose	0	0
Role location		
Metropolitan	17	94
Regional	1	6
Organisation		
Non-Government Organisation (NGO)	12	66
Government Organisation	6	33
Role setting		
Inpatient services	6	33
Community services	11	61
Unknown	1	6
Service delivery method		
Group work	0	0
Individual 1:1 service only	6	33
Both group work and individual 1:1 service	10	56
Unknown	2	11
Identify with ethnic or cultural group		
Yes	7	39
No	11	61
Identify as having a disability		
Yes	6	33
No	12	66
Peer support workers	17	94
Carer peer support workers	1	6
Completion of certificate IV in mental health Peer work		
Yes	2	11
No	3	17
Currently enrolled	2	11
Unknown	11	61

### Ethics

Ethical approval was provided for this research through the University of South Australia’s Human Research Ethics Committee (ID: 203551). Participants were provided with an information sheet outlining the research and requirements for participation. Informed written consent was received from each participant prior to participating in the individual interviews.

### Interviews

All interviews were conducted by the first author (VR) via videoconferencing or telephone and lasted between 20 and 80 min, with an average of 38 min. For the interview, participants were encouraged to place themselves in a comfortable environment they felt safe to share their experiences. Using a semi-structured interview guide created by the research team, questions focused on participant’s experience of integration into mental health care teams, what supports were provided by the organisation to facilitate integration and how, from their perspective, this may be improved or better supported. All interviews were recorded and professionally transcribed verbatim.

### Data Analysis

Data were analysed utilising thematic analysis and qualitative content analysis, as specifically described in Braun and Clarke (2006) and Mayring (2004). Familiarity with the data was established through the reading and re-reading of transcripts, approximately 3 times each before initial coding began. Coding was completed by the first author using NVivo 2020 v1.6 software, the first five transcripts were also coded by second author (ML) and code books were compared. The comparing of codes occurred after analysis the first five transcripts (28%) to prompt discussion around emerging themes from generalist and lived experience perspectives. The research team met regularly to discuss emerging themes within data, identify broad patterns and establish final themes. Recruitment for interviews continued until data saturation was reached and achieved after 18 interviews when no new or additional information or patterns were emerging from the data. Common themes derived from the data related to organisational culture, the role of leadership, and actions required by organisations to support integration, acceptance and sustainability of the peer support role.

Throughout the process of designing and conducting the study, Yardley (2015) principles for evaluating the quality and validity of qualitative data were used. Due to the exploratory nature of this research, reflexivity was prioritised to understand and explore how researcher motivations and perspectives may influence the interpretation of data and analysis. This was ensured through note taking during interviews and a continual sharing of perspectives and views between authors. The author team were varied in their positioning and approach to exploration of the data due to differing knowledge backgrounds including lived experience, social sciences and mental health nursing which allowed for greater unpacking and interpretation of the dataset.

## Results

Results of the interviews identified several areas for organisations to assist in promoting the acceptance and sustainability of the peer support workforce, as demonstrated in Table 3. These themes were established utilising thematic analysis and grouped through logical connectivity into areas of action including preparation, process and structural actions and cultural change for sustainability (Table 4). While peer worker experiences differed across working environments there was high consistency across perceived requirements to facilitate effective peer support practice. Each theme is presented and illustrated by participant quotes.

**Table 3:** Distribution of themes across interviews

Theme	Number of interviews theme identified	Number of references to theme
Robust induction to organisation	6	9
Training for existing staff	14	33
Clear referral pathways to peer support service	5	5
Consistent supervision and debriefing	9	16
Involvement of peer workforce	9	15
Leadership support	6	12
Professional development pathways	8	14

**Table 4:** Grouping of themes

Area of action	Theme
Preparation	- Education for existing staff on peer support role
	- Robust induction of peer workers to employing organisation
Process and structural changes	- Consistent supervision and debriefing for peer staff
	- Clear referral pathways into peer support service
Cultural change and actions for sustainability	- Regular involvement and consultation with peer workforce
	- Meaningful leadership support
	- Professional development pathways

### Preparation

Preparation refers to the early actions the organisation can take to facilitate a smoother transition of peer support workers into multidisciplinary healthcare teams. Participants suggested training and education for existing staff on the peer worker role, including focus and value of the role and how it is conducted. Some participants suggested a robust induction for new team members to the role including introduction of peer workers to the team, clear defining of role and responsibilities, provide opportunities for peer workers to share their story and discuss their role and how they can contribute with team members, in addition to other general organisational induction requirements. Finally, peer workers highlighted the need for appropriate recruitment to the role.

The need to educate staff and provide regular training on the peer support role and the application of recovery-orientated practices was noted in 15 of the overall 18 interviews.

“I think that more education around lived experience across the board……so that the value of lived experience can be embraced by all the staff” P13

Particularly, education around what the peer support role is, and how these fit within recovery-orientated frameworks and in practice.

“…people that don’t have an understanding of the role and are argumentative around it.” P7

“We all know what a clinician is and what they do. Whereas with peer work, there's less understanding. So there probably is more of a need for more awareness” P11

Participants suggested training and education to be delivered through additional modules to regular annual training delivered to all staff. Including education about the role and purpose of peer support work and how to work alongside and support/supervise those with a lived experience of mental distress. This form of training and education is suggested to be delivered at all levels of the organisation to ensure clarity, consistency and transparency of messaging.

“What we need to do is have orientation days, not just for peers, but for the whole workforce around lived experience” P15

“If the peer worker isn’t supported in the right way, they can just become another member of the team, and that power imbalance and the lack of compassion and empathy can become a thing, because they haven’t had the support.” P8

Appropriate recruitment was defined by several participants as recruiting people to a service who have prior lived experience of the service being delivered. An example of this would be to recruit an individual with a lived experience of hospitalisation associated with suicide related distress, employed in an acute or mental health unit of a hospital. This ensured the peer workers experience was related and beneficial to the individuals they support, in addition to providing insight to colleagues on what it is like to be on the receiving end of the service. Recruitment of this nature is also dependent on where an individual sits in their recovery journey and their willingness to work or re-engage with services where they experienced distress.

Finally, as part of the preparation identified to assist in the introduction of designated peer support roles is for organisations to provide a robust and inclusive induction to for new workers to the organisation. While it is noted that most organisations provide a general orientation to new workers, this study found the need for a more robust induction and introduction to the peer support role. Further, if the new worker is a starting in a designated peer support role, a thorough introduction of the worker and their role to their team and wider organisation is needed.

### Process and Structural Changes

Process and structural changes refer to shifts an organisation can make to general procedures and structures to adequately support and value peer workers in the workplace. This includes creating and embedding a referral process into peer support services alongside other supports offered to service users such as psychological services, social work or other community supports that assist in a person’s recovery journey. In several instances, participants noted not feeling valued or that their role may be viewed as tokenistic due to a lack of referrals from colleagues into peer support services. Referral pathways differ from organisation to organisation and for some, this was identified as a clear barrier to feeling included and part of the recovery team.

“I'm listening to peer workers who I'm working beside, who are distressed because of comments made in the workplace or them not getting referrals to their service because they're deemed not important enough.” P17

Other participants who noted lack of referral pathways identified a potential lack of understanding of the role by colleagues, leading to reduced promotion of peer services to service users and fewer referrals.

“If the client’s know what peer workers do, they’re more likely to want them in the service” P6

This may also be attributed to service users’ lack of knowledge of the peer support role, prompting need for clinicians to educate the individual on all options available for support and assistance in their recovery journey.

“I think this is in general, a lack of understanding around what we actually do, what a session actually looks like” P11

This emphasises the need for process change within teams and organisations to define clear and effective referral pathways into peer support services. Further process change noted by several participants was the need for consistent and meaningful professional supervision provided to peer workers. Providing a safe space for peer workers to reflect on their practice, discuss interpersonal relations at work, consider alternatives linked to difficult or challenging interactions, in addition to receiving workplace supports from line management was deemed essential in peer workers feeling accepted and valued as part of the team.

“Checking in with peer specialists in the early days, just to make sure that everything’s okay. A bit of support.” P10

Several participants noted a lack of formal supervision within their peer support roles, or receiving supervision from someone with little knowledge or compassionate understanding of the peer support role. Several participants noted the importance of psychologically safe encounters through supervision and debriefing alongside a senior peer worker either within or outside the organisation to discuss approaches to utilising their lived experience or provide professional development.

“Debriefing because myself and the other peer workers have mental illness. So if we come back from a client visit or if something happens in a group, the team rallies around us peer workers and says, do you want a debrief about this, and the managers as well” P5

“I think that would be really beneficial, having a peer worker… or a lived experience person in a management role and running the supervisions.” P18

Further to referral pathways and consistent professional supervision, participants suggest structural changes to how peer support work and expertise are validated through higher pay rates. This is through higher pay awards for peer workers, particularly if they had completed formal training, incentivising engagement with nationally accredited and recognised training qualifications in peer support.

“Means that we are already kind of intrinsically validated by the organisation to say that, all right, well we value you enough to pay you this pay point” P1

Higher pay awards were noted by several participants in this study as a means of validating the service peer support professionals provide, in addition to prompting feelings of value to the organisation.

### Culture for Sustainability

The final area identified was the introduction of practices to shift counterproductive and entrenched mindsets regarding expertise and inclusion within organisational cultural norms. Cultural shifts such as involvement of peer workers in decision making and policy development, meaningful leadership support and follow through and avenues for professional development and career pathways.

Several participants highlighted the need for the involvement and consultation from lived experience worker expertise in relation to service development.

“To provide that voice at high levels. So, decisions are made always with the lived experience recovery-oriented trauma-informed focus.” P17

“I think, if you're going to try to create a service that works, I mean, why not ask people that have been accessing services?” P15

This was also referred to in a broader sense of providing opportunity for consumers to be involved at all levels of policy development and governance.

“Having lived experience, people at the table, and so including lived experience people in the decision-making processes…. consulting with lived experience people at all levels” P11

“We need someone with lived experience to sit at an executive level to try to help the policies and the work.” P3

“Involving peer workers in as much as possible of the decision making that happens in the team and development of policies” P10

Meaningful and open support from organisational leadership was identified by several participants as crucial to facilitating acceptance of peer support and other designated lived experience roles within multidisciplinary mental health teams:

“I think leadership is vitally important and crucial. Leadership has the ability to implement direction, set culture, uphold values, set an example - there’s so much that a leader can do.” P7

“Management keep the recovery language strong amongst all your team, like amongst all disciplines” P9

“This is critical that the management has the experience and expertise to actually work with this framework of – there could be a real growth and learning experience and richness within that.” P7

Providing workers with career progression and opportunities for professional development was frequently discussed by participants to promote value of peer support work throughout organisations.

“I’ve never been paid as someone who’s a mentor or a team leader… so if I had the opportunity I would definitely go for the job.” P6

“Get senior peer roles and peer supervision…. and also to provide a pathway, because I started off at what’s called level 1 and now I’m level 2 and there’s nowhere to go.” P8

Further, to support peer workers to develop and promote career progression through development of peer leadership positions.

“I also think that there should be lived experience people in all tiers of an organisation” P12

“I think for senior lived experience roles to be more adopted in workplaces so that peers can get peer supervision, just like clinicians get clinical supervision.” P15

## Discussion

This study provides detailed insight of peer worker experiences and perspectives for successful integration into mental health service teams. The findings from this research suggest barriers remain in the integration of peer support roles in mental health service organisations. Findings, whilst consistent with research investigating key facilitators and barriers for improving integration of peer support workforces in mental health care (Byrne et al., 2021a; Ibrahim et al., 2021), build upon and advance this earlier work by identifying and establishing key areas of action to support organisational change. These include practical strategies for organisations to consider when implementing peer support roles. These findings are emphatic in the need for organisations to wholly commit and provide effective and meaningful support for peer work practice.

The introduction of peer support roles within organisations is noted as a fundamental step in services moving towards greater recovery-orientated practice and values (Franke et al., 2010). Ensuring existing staff are prepared and understand the role, are supportive and have confidence in the service is integral to facilitate the roles acceptance (Berry et al., 2011; Byrne et al., 2019b; Chinman et al., 2006, 2010). Recruitment of appropriate expertise for the services delivered ensures peer workers have relevant knowledge of the service, a peer work skillset in addition to their lived experience of recovery (Byrne et al., 2021a; Jacobson et al., 2012). Providing training to existing staff and affording opportunities to engage and ask questions of peer workers may also encourage buy-in and acceptance (Chinman et al., 2008b; Davidson et al., 2012; Gates et al., 2010). However, education and training is only one element of the required whole-of-organisation commitment to cultural change for shifts in attitudes and behaviours toward adoption of recovery-orientated practice and acceptance of peer support work (Johnson et al., 2016).

Previous research has discussed the need for ‘whole-of-organisation’ change, noting that fitting peer support services into existing structures and organisational procedures as being insufficient and diverging from intended recovery-orientated practices (Byrne et al., 2021a; Gillard et al., 2016). The findings indicate that structural and process changes need to be initiated by organisations, including consistent and ongoing supervision, referral pathways into the service and higher pay to validate peer knowledge and services provided. This is supported in literature, indicating that regular peer led supervision from an empathic and understanding supervisor provides required support for transitioning into the role, allowing opportunities to debrief, share strategies and develop skills, knowledge and expertise (Davidson et al., 2012; Delman & Klodnick, 2017; Repper & Watson, 2012).

Creating pathways for professional development was noted across participants as integral for career progression and contributing to the sustainability of peer support roles. Clear progression pathways reduce potential for implementing tokenistic or generic peer roles within organisations, and provides clarity for possible career progression and development beyond senior practitioner roles. Supported by literature, this emphasises the need for peers in leadership and supervisor positions, creating opportunity to provide involvement and coproduction at all levels of an organisation (Byrne et al., 2018a, 2018b; Gates et al., 2010; Mancini, 2018; Nestor & Galletly, 2008).

Clear referral pathways for people into peer support services facilitates the inclusion of peer workers into a team, promotes a culture of acceptance and ensures peer workers feel valued for the services they provide (Gates et al., 2010; Shepardson et al., 2019). Increasing the promotion of peer work services enables greater understanding of the service, encourages acceptance of the role in multidisciplinary teams and contributes to its sustainability. Consideration should also be given as to how organisations promote peer support services externally to potential service users and public audiences more broadly.

Workplace culture has been noted through literature as a common barrier for the effective implementation and integration of peer workers, particularly the influence leadership has on facilitating a positive and productive organisational culture (Byrne et al., 2016; Ibrahim et al., 2021; Vandewalle et al., 2016). In many organisations, leadership has the ability to take a “top-down” approach and implement strategies to assist with cultural change and facilitate the integration of new employees into service teams (Byrne et al., 2019a, 2019b). Leaders play a crucial role in legitimizing peer support services by promoting and prompting the use of inclusive recovery-orientated language (Zeng & McNamara, 2021), ensuring effective organisation wide problem solving, lifting the visibility of roles and evaluating achievements (Mulvale et al., 2019). Despite this, recent research indicates leadership support alone is not sufficient and organisations should take a ‘whole-of-organisation’ change approach to support acceptance and sustainability (Byrne et al., 2021a, 2021b). This further highlights the importance of effective change across multiple levels within an organisation including worker mindsets and culture, structures and practices, resource flows, relationships and policy to support overall systems change (Loughhead et al., 2021). This is consistent in industry with research acknowledging the importance of organisational preparedness as being essential for organisational change to improve service delivery outcomes and standards (State of Victoria, 2013).

Similarly, the consistent use of recovery language in services and throughout organisational messaging promotes adoption of recovery-orientated practice which supports use of lived experience expertise and peer support. With the field of mental health undergoing significant paradigm shifts with adoption and integration of recovery-orientated thinking and practice, much consideration is required for the impact or re-thinking of the meaning of social inclusion, language, empowerment and overall recovery in mental health (Glover, 2012). Providing organisations and service workers with tools that enable and facilitate productive and inclusive working environments is crucial to support such fundamental paradigm shifts.

Results of this study indicate that the successful integration of peer support workers into mental health teams requires a multi-layered approach to organisational development. This is consistent with findings from earlier studies on the experience of workers and required organisational shifts (Byrne et al., 2021a; Glover, 2012; Mancini, 2018; Piat et al., 2017). Despite this focus on the integration of peer workers, an industry focus on integration alone is not sufficient to alleviate the acknowledged challenges as established in literature. Future research may benefit from investigating how organisational leaders can define the purpose and scope of the peer role within the organisation and provide clarity to peer support workers in regard to their key duties and responsibilities. As per the perspective of participants, change is required at multiple levels throughout organisations to ensure that consistent and coordinated strategies are implemented to facilitate better understanding and ultimately, valuing of peer worker expertise. Given what is known about required organisational change, this study adds value by emphasising a focus on the following considerations:

- Robust preparation of teams and workspaces to reduce instances of ‘othering’ or stigmas or assumptions to peers.
- Evaluating organisation ‘readiness’ for structural and cultural changes and implementing strategies to positively assist with this change including training for the wider workforce and comprehensive orientation for new peer support workers
- Taking consistent action to generate a culture that values lived experience expertise through training and education, consistent of use of recovery language, commitment and inclusion at all levels of the organisation

## Limitations

This research may be limited due to smaller sample size of included participants (*n* = 18). Future research may attempt to include a larger number of participants. Potential for selection bias may also be a limitation due to participants’ self-nomination for inclusion in the study. The current findings are limited to peer support workers and may not be generalizable to include experience of all other designated lived experience roles.
